# Icariin Ameliorates Streptozotocin-Induced Diabetic Retinopathy *in Vitro* and *in Vivo*

**DOI:** 10.3390/ijms13010866

**Published:** 2012-01-16

**Authors:** Hua Xin, Feng Zhou, Tao Liu, Guang-Yong Li, Jing Liu, Zhe-Zhu Gao, Guang-Yi Bai, Hong Lu, Zhong-Cheng Xin

**Affiliations:** 1 Department of Ophthalmology, Beijing ChaoYang Hospital, Capital Medical University, Beijing 100043, China; E-Mail: xinly520@hotmail.com; 2 Andrology Center, Peking University First Hospital, Peking University, Beijing 100034, China; E-Mails: zhoufeng0319@163.com (F.Z.); lyittle@163.com (T.L.); guangyongli1979@hotmail.com (G.-Y.L.); liujing827@yahoo.cn (J.L.); gaozhezhu@gmail.com (Z.-Z.G.); bgy73kr@hanmail.net (G.-Y. B.)

**Keywords:** icariin, streptozotocin, diabetes, retina, diabetic retinopathy

## Abstract

This study investigated the effect of Icariin (ICA) supplementation on diabetic retinopathy (DR) in a streptozotocin-induced diabetic rat model system. Fifty Sprague Dawley rats were randomly distributed into a control group and a streptozotocin-induced diabetes group. Diabetic rats were randomly divided into two groups; one group received ICA 5 mg/kg/day for 12 weeks by oral gavage; the other group received saline gavage as a placebo. Retinal morphological changes, endothelial markers (RECA), collagen IV (Col-IV), vascular endothelial growth factor (VEGF), and neuropathic changes (*Thy-1* and *Brn3a* expression) of the retinal ganglion cells (RGCs) were investigated. The effects of ICA at various concentrations (0, 10^1^, 10^2^, 10^3^ nmol/mL) on neurite growth were investigated also in retinal ganglion cells (RGC) cultured from both diabetic and normal animals. Numerous pathological changes (deceased expression of *RECA*, *VEGF*, *Thy-1*, and *Brn3a* as well as decreased Collagen IV and Müller cell content) were noted in the retinal vessels of diabetic rats; these changes were attenuated in diabetic animals that received ICA. ICA enhanced neurite growth in RGC from both normal rats and diabetic rats in a dose dependent fashion. ICA may be useful in the treatment of diabetic retinopathy. Further investigations are indicated.

## 1. Introduction

Diabetic retinopathy (DR) is a leading cause of post-natal blindness and one of the most common complications of diabetes [1,2]. Epidemiologic studies indicate that, most patients with type 1 diabetes or insulin-dependent patients with type 2 diabetes will experience retinopathy within 20 years of diagnosis, even patients with non-insulin-dependent type 2 diabetes have an approximately 50% prevalence of retinopathy within 20 years of diagnosis [3].

Microvascular lesions such as microaneurysms, increased vascular permeability caused by the breakdown of the blood-retinal barrier (BRB), and capillary dropout are thought to be key causes of DR [4–6]. The sole purpose of retinal circulation is to support the metabolic demands of the inner retinal neurons and glial; these cells may also be damaged by the diabetic state. Retinal ganglion cells (RGC) are the sole output neurons from the eyes, assuming the critical role of transmitting visual signals to the higher visual center at the brain cortex before signal processing. Thus, neuronal, glial, and specifically RGC dysfunction may occur in unison with blood flow abnormalities and often before the appearance of overt microvascular damage [7].

The Diabetes Control and Complications Trial (DCCT1993) investigated the effect of hyperglycemia in type 1 diabetic patients, as well as the incidences of diabetic retinopathy, nephropathy, and neuropathy. Intensive diabetes management with three or more daily insulin injections or a continuous subcutaneous insulin infusion decreased the risk of progression of retinopathy by 54% (95% CI 39–66), While insulin therapy has been shown to be efficacious in preventing progression of retinopathy in diabetes, problems with administration and some variability in efficacy warrants a need for continued development of new therapies for diabetic retinopathy.

**Scheme 1:**
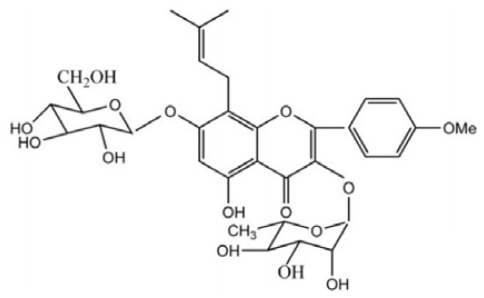
The chemical structures of Icariin.

Traditional Chinese Medicine (TCM) has been utilized for centuries and has a unique therapeutic role in the treatment of many human diseases. Many herbal therapies used in TCM contain compounds with demonstrablebiological activities in both experimental and clinical settings. Icariin (ICA, C_33_H_40_O_15_, molecular weight: 676.67) is thought to be the principal active moiety of Epimedii herba (Scheme 1).

Xin and colleagues, in a pharmacological study, reported that icariin was a cGMP-specific phosphodiesteras5 (PDE5) inhibitor; the selectivity of icariin for PDE5 over PDE4 is approximately 167-fold [8] and ICA has been shown to bind to the active catalytic domain of PDE5A1, similar to the activity of the erectogenic medication Sildenafil [9]. Liu *et al*. have reported that icariin improves erectile function and nitric oxide synthase (NOS) expression in the corpora cavernosa of castrated rats without influence on serum testosterone levels [10]. Shindel *et al*. reported that icariin enhanced growth of neurite from cultured major pelvic ganglia (MPG) fragments and improved penile hemodynamics in rats with injury to the cavernous erectile nerves [11]. Icariin has also shown efficacy in treatment of impaired penile hemodynamics in rodents with streptozotocin-induced diabetes by regulation TGF-β-Smad signaling pathway [12]. Also Icariin can evidently relieve renal damage in rats with diabetic nephropathy induced by streptozocin, which might be related to modulating the expression of collagen IV and TGF-beta1 protein [13].

We are not aware of any studies on ICA for management of complications related to diabetic retinopathy. Given the apparent neurologic and vascular benefits of ICA, we hypothesized that ICA supplementation might help to ameliorate pathological changes of the retina in rats with experimentally induced diabetes. We investigated the effects of ICA on regulation *RECA* and *Col-IV* expression in retinal endothelium and *Thy-1* and *Brn3a* expression in RGC from rats with Streptozotocin-Induced Diabetes compared to controls.

## 2. Results

### 2.1. Metabolic and Physiological Variables

The initial body weight and initial fasting serum glucose level in normal rats and diabetic rats were not significantly different (Table 1, *P* for difference > 0.05). At the end of study mean body weight was decreased significantly, and final fasting serum glucose level increased in diabetic rats compared to sham controls (Table 1, *P* for difference < 0.01). ICA treatment did not significantly alter blood glucose or weight in diabetic rats (*P* > 0.05) (Table 1).

### 2.2. Morphological Changes of the Retina

**Figure 1:**
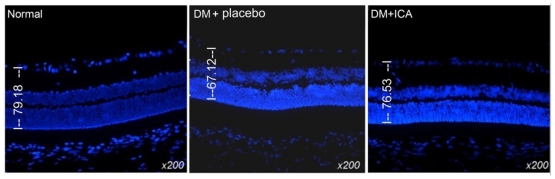
The effect of icariin on morphological changes of disbetic retinopathy. The notable morphological changes were observed in diabetic retina, however, these changes were improved by ICA treated diabetic retina and the average thickness of retina in different groups.

Comparing placebo treated diabetic to control animals, numerous Morphological changes were observed in inner nuclear layer (INL), outer nuclear layer (ONL), retinal ganglion cells (RGCs), and the intensity and number of bipolar cells in the INL and ONL. RGC were considerably reduced in diabetic group as compared with those of the controls. The thickness of the basal membrane in diabetic group was significantly decreased (79.18 ± 5.4 um *vs*. 67.12 ± 4.8 um). In the ICA group, the thickness of basal membrane was 76.53 ± 6.1 um (*P* < 0.05). The morphological structure of the retinal specimens was qualitatively better in the ICA treated group (Figure 1).

### 2.3. *RECA*, *Col IV* and *VEGF* Expression in Microvasculature of the Retina

**Figure 2:**
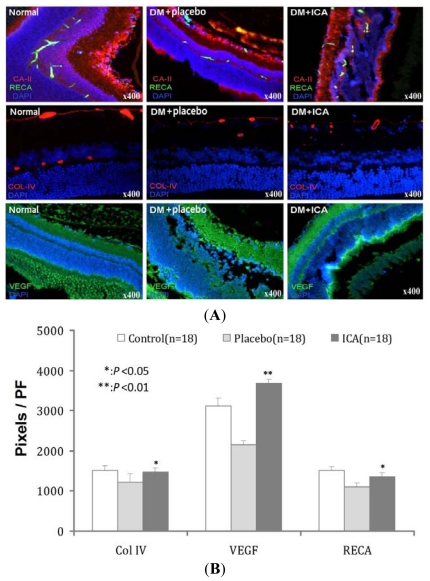
The effects of ICA on Col IV, RECA and VEFG expression micro blood vessels in diabetic retina. *RECA*, *Col-IV*, and *VEGF* were used to check the retinal microvessls. *RECA* and *Col-IV* clearly demonstrated the distribution of retinal vessels. As described in the text, DM decreased the blood vessels density in retina while ICA improved these conditions. It is noted that ICA improved the expression of *VEGF* significantly.

Retinal blood vessels are clearly defined in retinal pigment epithelium (Figure 2). Collagen IV expression was less in the diabetic retina, consistent with thickening of the micro-vessel basement membrane. Basement membrane thickening was less in the ICA treated diabetic group compared to placebo-treated diabetic animals. ICA treated animals also had greater expression of *VEGF* and microvessel density (Table 2, Figure 2).

### 2.4. Effects of ICA on *Thy-1* and *Brn3a* Expression in RGCs of DR

**Figure 3:**
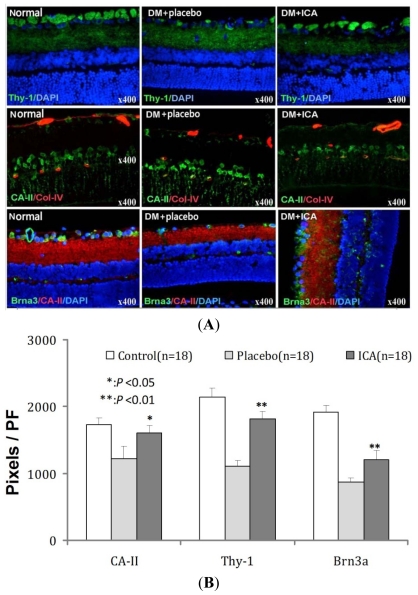
The effects of ICA on RGCs *Thy-1* and *Brna-3* and *CA II* expression in Diabetic retina. *Thy-1* and *Brn3a* were used to detect the RGCs (in up panel and lower panel), CA-II was used to detect the Müller cells (in middle panel). Diabetes significantly decreased the RGCs and increased Müller cells in the retina, while ICA improved both pathological situations.

*Thy-1* and *Brn3a* expression in diabetic retinas were significantly decreased in the inner nuclear layer (INL), outer nuclear layer (ONL), retinal ganglion cells (RGCs) compared to sham control retinas. Diabetic rats treated with ICA had greater expression of Thy-1 and Brn3a relative to placebo-treated diabetic animals (Figure 3). More importantly, those cells were re-organized well along the retina. Müller cells support neuronal activity and the integrity of the blood-retinal barrier, whereas gliotic alterations of Müller cells under pathological conditions may contribute to retinal degeneration and edema formation [14]. CA-II was used to detect the Müller cells (in middle panel); it is notable that there was a significant difference in retinal Müller cells in rats treated with ICA. (Table 3, Figure 3)

### 2.5. Effects of ICA on RGC Neurite Outgrowth from Retina *in Vitro*

**Figure 4:**
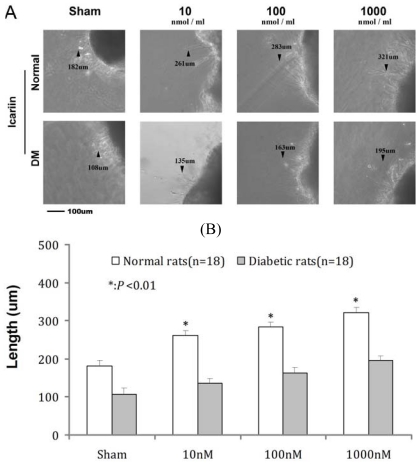
The neurites sprout from retina after stimulation with ICA in normal rats and diabetic rats. Paired comparisons were made between retina derived from normal control and DM rats at the treatment of 0, 10, 100 and 1000 nmol/mL ICA, all retina treated with ICA had significantly longer average neurite length in different dose of ICA in normal rats and diabetic rats when compared to DM group.

Neurite outgrowth was measured in cultured RGC from diabetic and normal rats. Paired comparisons were made between retina derived from normal control and DM rats at the treatment of 0, 10, 100 and 1000 nmol/mL ICA at the 72 h time point, all retina treated with ICA had significantly longer average neurite length when compared to DM group. (Table 4, Figure 4)

## 3. Discussion

In the present study, we provided evidence that icariin may be of benefit in the DR prevention in diabetes. Icariin treated diabetic rats had significantly greater microvessel density, thinner basement membranes, greater expression of endothelial and ganglia markers, compared to the control group. Importantly, there was no significant difference in metabolic and physiological variables between the diabetic control and ICA treated group, implying that these effects showed independent of serum glucose level.

Our previous study of ICA improving diabetic erectile dysfunction demonstrated that ICA improves erectile hemodynamics in castrated animals and animals with STZ-induced diabetes without significantly influencing testosterone or glucose levels, and ICA influenced the nitric oxide synthase (endothelial and neuronal) in microvascular complications of diabetes mellitus [12]. It is suggested that the effect of ICA in diabetes is not hyperglycemic control but rather some other mechanism that is yet to be elucidated.

Among the pathological changes that occur early and linked causally to the development of retinopathy in diabetes are inflammation, altered extracellular matrix (ECM) gene expression, and premature demise of retinal capillary and ganglion cells [7,15]. Other potentiall important mediators include advanced glycosylation end products and its receptor (*AGEs*/*RAGEs*), oxidative stress by oxygen free radical, inflammation, fibrosis, neuropathy and hypogonadism [16,17] It is not yet clear which of these components are most important for disease initiation and development of DR. Identification of the principal effectors of DR I of import as this may permit selection of ideal therapeutic targets.

Retinal microvascular changes in DR might be related to hyperglycemia-induced intramural pericyte death and thickening of the basement membrane. This may lead to incompetence of the vascular walls and disruption of the blood-retinal barrier [18]. In our study, retinal microvessell density is significantly decreased in diabetic animals compared to normals.

Endothelial deterioration and neuropathy are also important pathological process in diabetic retinopathy models [19]. Intracellular hyperglycemia has been linked to an overproduction of ECM; hyperglycemia may also decrease production of trophic factors for endothelial and neuronal cells, and the intracellular oxidative stress in endothelial cells plays a key role in endothelial dysfunction [20]. Together, these changes lead to neuropathy and endothelial lesions [21,22]. Collagen IV is one of the major components in the blood vessel basement membrane. The expression of collagen IV during diabetes has been extensively studied. It has been reported that collagen IV is highly expressed in the diabetes and related to many diabetic pathological disorders [23]. The major expression of *VEGF* is related with the revascularization and a number of clinical studies have shown a strong correlation between increases in intraocular *VEGF* concentration and the development of proliferative diabetic retinopathy. Interestingly, our results demonstrate that *Col IV*, *RECA* and *VEGF* expression the retina were decreased in diabetic rats compared to normal controls.

Cultured retinal ganglion is an important and almost an indispensable tool for the study of retinal visual physiology and pathophysiology, and it may easily evolve as state-of-art technology for studying the inter-cellular or intra-cellular processes associated with various retinopathies and neuropathies [24]. In our study ICA appeared to enhance neurite length in both normal and diabetic rat retinas. This effect was dose dependent, with greater concentrations leading to greater mean neurite length.

Our data are limited in that functional assay of visual acuity was not performed in the various treatment groups. This sort of investigation is challenging in rodent subjects but should be a consideration for future work. Future studies on ICA in DR should also incorporate different treatment time courses as well as additional mechanisms such as *AGEs*/*RAGEs*, oxidative stress, growth factor, *etc*.

## 4. Materials and Methods

### 4.1. Animal and Treatment

A total of 50 male 8 week-old Sprague-Dawley rats weighting 200–250 g were used in this study. The experiments were approved by the Institutional Animal Care and Use Subcommittee at our university. Rats were fasted for 16 h, and 38 rats (diabetes mellitus group, DM) were injected intraperitoneally with freshly prepared streptozocin (STZ, Sigma Chemical Co, St Louis, MO, USA) (60 mg/kg) and 12 rats (sham group) injected vehicle (0.1 mol/L citrate-phosphate buffer, pH 4.5) according to the references [25,26]. Blood glucose levels were monitored 72 h later after STZ or vehicle injection, at regular intervals of every week throughout the study, and immediately prior to euthanasia. Blood samples were obtained by tail prick, and blood glucose concentration measured using a blood glucose meter (B.Braun, Germany). Only rats with fasting glucose concentrations (≥300 mg/dL) were included in the DM group. A total 36 rats were developed into diabete and been divided into 2 groups and fed with 50:50 mix of normal saline and dimethyl sulfoxide (DMSO, placebo, *N* = 18) or Icariin (ICA) in 50% DMSO (ICA, *N* = 18) at 5 mg/kg (the best effective dose in our previous study [12]) daily doses respectively for 12 weeks. Rats in the sham group received standard husbandry care, gavage feeded with PBS, but were not treated with STZ. At the end of 12 weeks rats were euthanized and retinas harvested for histological and molecular study.

### 4.2. Selection of Tissue Markers

Rat endothelial cell antigen protein (*RECA*) and vascular endothelial growth factor (*VEGF*) are important biomarkers in vascular biology. Collagen IV (*Col-IV*) is an important marker of basement membrane competence in blood vessels and the relationship between *Col-IV* and diabetes has been extensively studied [27,28].

Important markers for RGC damage include oxidative stress, *Thy-1* (a surface glycoprotein of the immunoglobulin superfamily specifically expressed in RGC), and the transcription factor *Brn3a* (a transcription factor specifically expressed in cells of the developing mammalian nervous system) [29–31].

### 4.3. Immunofluorescence Stain

After euthanasia, the retina was harvested and immersed in neutral buffered formalin containing 4% formaldehyde for a period of 6 h, embedded in liquid nitrogen. Sections of 8 μm thickness were cut using a freezing microtome.

For immunofluorescence, the tissues were cryoprotected in sucrose, frozen and sectioned at 8 μm in a cryostat. Slides were incubated successively with blocking solution. The tissue sections were incubated with primary antibody to Rat Endothelial Cell antigen (RECA, Abcam, Cambridge, MA, USA; 1:200), Collagen IV antibody (Col-IV, Abcam, Cambridge, MA, USA; 1:800), Vascular Endothelial Growth Factor (VEGF, Abcam, Cambridge, MA, USA; 1:400), Thy-1 cell surface antigen (Thy-1, Santa Cruz Biotechnology, CA, USA; 1:200), Brain-specific homeobox/POU domain protein 3A (Brn3a, Abcam, Cambridge, MA, USA; 1:400), Carbonic Anhydrase II (CA-II, Abcam, Cambridge, MA, USA; 1:400). After the hybridization of secondary antibodies, and DAPI staining for the cell nucleus, the sections were observed at the fluorescence microscope (Leica DM 6000 Laser Station). Semiquantitative analysis was performed to evaluate the intensity of *RECA*, *Col-IV*, *VEGF*, *Thy-1*, *Brn3a*, and *CA-II* staining by the use of Image pro plus software 6.0 (Media Cybernetics, Silver Spring, MD, USA).

### 4.4. Retina Ganglion Cell Tissue Culture *in Vitro*

The retinal ganglion tissue of sham group and DM placebo group (untreated) (*n* = 18) were cultured *in vitro*, stimulation with different concentration of ICA and the length of neurite outgrowth was measured.

Each retinal tissue specimen was divided into two sections. After PBS rinsing, the freshly dissected retina ganglion fragment was placed on a coverslip, to which a 14 μL drop of growth-factor-reduced Matrigel™ (Becton Dickinson, Mountain View, CA, USA) had been added and kept in liquid form using a cold 35-mm plastic culture dish on ice. The growth factor reduced Matrigel™ was polymerized (5-min incubation at 37 °C) and 2 mL of serum-free RPMI-1640 (Cell Culture Facility, University of California, San Francisco, CA, USA) added. Tissue fragments from the control and diabetic group were treated at various concentrations of ICA for 3 days (0, 10, 100, 1000 nmol/mL ICA). Ganglial cultures were maintained at 37 °C in a humidified atmosphere with 5% CO_2_. Photographs of neurite growth at 6 days were captured using a Nikon DXM 1200 digital still camera attached to Leica Laborluxmicroscope and ACT-1 software (Nikon Instruments Inc., Melville, NY, USA). Digital images were analyzed using Image-Pro Plus software to determine the longest neurite length per specimen. Mean maximal neurite length was calculated for the control and diabetic group by averaging the longest neurite length from each individual specimen.

### 4.5. Statistical Analysis

Results are expressed as means ± standard deviation. One-way ANOVA followed by Bonferroni multiple comparisons’ test was used to evaluate whether differences between groups were significant. All calculations were performed using SPSS statistical software (version 13.0, SPSS, Chicago, IL, USA). Probability values of less than 5% were considered significant.

## 5. Conclusions

ICA may be useful in the management of DR by modulating both *RECA* and *Col-IV* expression in retinal microvessls and *Thy-1* and *Brn3a* expression in RGC (5 mg/kg/day p.o.). These effects appear to occur independently of serum glucose levels.

## Figures and Tables

**Table 1:** The effects of Icariin (ICA) on Metabolic and physiological variables.

Variable	Diabetic ED Model		Sham (*n* = 12)

	placebo (*n* = 18)	ICA (*n* = 18)	
Initial weight(g)	253.5 ± 7.2	251.3 ± 11.9	264.1 ± 12.4
Final weight(g)	247.6 ± 12.2 **	254.2 ± 15.2 **	568.2 ± 16.4
Initial fasting glucose(mg/dL)	107.1 ± 4.8	102.4 ± 5.3	115.2 ± 6.1
Initial postprandial glucose(mg/dL)	128.2 ± 8.2	119.5 ± 11.4	131.9 ± 14.6
Final fasting glucose(mg/dL)	382.4 ± 31.4 **	392.6 ± 32.4 **	102 ± 13.6
Final postprandial glucose(mg/dL)	485.2 ± 31.7 **	516.7 ± 23.6 **	136.1 ± 11.8

Values are the mean values (±standard deviation)

** *P* < 0.01 compared with the placebo group.

**Table 2:** The effects of ICA on *Col IV*, *RECA* and *VEFG* expression micro blood vessels in diabetic retina.

Groups	*Col IV*	*VEGF*	*RECA*
Normal	1510 ± 121	3128 ± 181	1521 ± 95
DM + placebo	1227 ± 198	2145 ± 97	1103 ± 101
DM + ICA	1466 ± 101 *	3678 ± 110 **	1359 ± 87 *
*P* value	<0.05	<0.01	<0.05

*RECA*, *Col-IV*, and *VEGF* were used to check the retinal microvessls. Values are the mean values (±standard deviation) from *N* = 18 animals per group.

* *P* < 0.05

** *P* < 0.01 compared with the placebo group.

**Table 3:** The effects of ICA on RGCs *Thy-1* and *Brna-3* and *CA II* expression in Diabetic retina.

Groups	*CA-II*	*Thy-1*	*Brna3*
Normal	1728 ± 101	2138 ± 143	1923 ± 98
DM + placebo	1219 ± 192	1106 ± 87	870 ± 65
DM + ICA	1601 ± 121 *	1820 ± 110 **	1213 ± 126 **
*P* value	<0.05	<0.01	<0.01

*Thy-1* and *Brn3a* were used to detect the RGCs (in up panel and lower panel), *CA-II* was used to detect the Müller cells (in middle panel). Values are the mean values (±standard deviation) from *N* = 18 animals per group.

* *P* < 0.05,

** *P* < 0.01 compared with the placebo group.

**Table 4:** The neurites sprout from retina after stimulation with ICA in normal rats and diabetic rats.

Groups	Sham	10 nM	100 nM	1000 nM
Normal	182 ± 4.1 **	261 ± 2.1 **	283 ± 3.3 **	321 ± 4.6 **
DM	108 ± 5.3	135 ± 2.3	163 ± 4.1	195 ± 3.8
*P* value	<0.01	<0.01	<0.01	<0.01

Paired comparisons were made between retina derived from normal control and DM rats at the treatment of 0, 10, 100 and 1000 nmol/mL ICA at the 48 h time point. Values are the mean values (±standard deviation) from *N* = 12 animals per group.

** *P* < 0.01 compared with the DM group.
